# Postprandial glucose metabolism and SCFA after consuming wholegrain rye bread and wheat bread enriched with bioprocessed rye bran in individuals with mild gastrointestinal symptoms

**DOI:** 10.1186/1475-2891-13-104

**Published:** 2014-11-04

**Authors:** Jenni Lappi, Hannu Mykkänen, Knud Erik Bach Knudsen, Pirkka Kirjavainen, Kati Katina, Jussi Pihlajamäki, Kaisa Poutanen, Marjukka Kolehmainen

**Affiliations:** Institute of Public Health and Clinical Nutrition, University of Eastern Finland, PO Box 1627, FIN-70211 Kuopio, Finland; Department of Animal Science, Molecular Nutrition and Cell Biology, Aarhus University, 8830 Tjele, Denmark; Department of Environmental Health, National Institute for Health and Welfare, PO Box 95, FIN-70701 Kuopio, Finland; Department of Food and Environmental Sciences, Food Science, University of Helsinki, P.O. Box 66, FIN- 00014 Helsinki, Finland; VTT Technical Research Centre of Finland, Espoo, FIN-02044 VTT Finland

**Keywords:** Postprandial, Glucose metabolism, Short chain fatty acids, Rye, Wheat, Wholegrain, Bran, Bioprocessing, Gastrointestinal symptoms

## Abstract

**Background:**

Rye bread benefits glucose metabolism. It is unknown whether the same effect is achieved by rye bran-enriched wheat bread. We tested whether white wheat bread enriched with bioprocessed rye bran (BRB + WW) and sourdough wholegrain rye bread (WGR) have similar effects on glucose metabolism and plasma level of short chain fatty acids (SCFAs).

**Methods:**

Twenty-one (12 women) of 23 recruited subjects completed an intervention with a four-week run-in and two four-week test periods in cross-over design. White wheat bread (WW; 3% fibre) was consumed during the run-in, and WGR and BRB + WW (10% fibre) during the test periods. A meal test providing 51/33/11 E % from carbohydrates/fat/protein was conducted at the end of each period. Fasting and postprandial plasma samples were analysed for glucose, insulin, and SCFA.

**Results:**

Glucose and insulin responses and plasma concentrations of SCFAs to the meal test were similar between the WGR and BRB + WW periods. When compared to the WW period, postprandial insulin concentration at 120 min was lower (p = 0.023) and the first-phase insulin secretion improved (p = 0.033) only after the WGR period, whereas postprandial concentrations of butyrate (p < 0.05) and propionate (p = 0.009) at 30 min increased during both rye bread periods.

**Conclusions:**

Beneficial effects of WGR over white wheat bread on glucose and SCFA production were confirmed. The enrichment of the white wheat bread with bioprocessed rye bran (BRB + WW) yielded similar but not as pronounced effects than WGR when compared to WW alone. Postprandially measured glucose metabolism and concentrations of SCFAs provided additional information along with fasting measurements.

## Background

Potential benefits of rye bread intake over wheat bread regarding glucose metabolism has been demonstrated during the past 15 years. A single meal of rye bread has been repeatedly shown to reduce postprandial insulin response as compared with a single meal of white wheat bread [1–3], suggesting a beneficial effect on glucose metabolism. However, the data from longer-term interventions is less clear. Consumption of wholegrain rye bread enriched with rye bran daily for eight weeks improved the first-phase insulin secretion measured by the frequently sampled intravenous glucose tolerance test (FSIGT) in healthy postmenopausal women [4]. Similar enhancement in the first-phase insulin secretion measured by the oral glucose tolerance test (OGTT) was observed in subjects with the features of metabolic syndrome consuming rye bread and pasta daily for 12 weeks [5]. In contrast to these findings, no difference was observed in the first-phase insulin secretion or insulin sensitivity in subjects with metabolic syndrome consuming rye bread or a combination of wholegrain wheat and rye foods daily for 12 weeks, as compared to those consuming refined wheat foods [6, 7].

Reduced glucose responses to a standardized meal test or OGTT have been observed in second-meal studies where a single evening meal containing high-fibre barley kernels was served to healthy subjects ten hours before the test [8–11]. Furthermore, plasma butyrate and/or propionate concentrations were found to be increased after the standardized test breakfast when the preceding evening meal contained barley when compared with the evening meal with white wheat bread [8, 10, 12]. These data suggest that butyrate and propionate that are produced by intestinal fermentation of grain fibre after a meal with whole grain cereals are associated with improvement of the later postprandial glucose metabolism. Regular consumption of high-fibre rye bread could also affect postprandial glucose metabolism via production of short chain fatty acids (SCFAs) after a meal test, but to date, there are no studies to support this.

Wholegrain foods are protective against type 2 diabetes and cardiovascular diseases [13], but the protective factor in wholegrain foods may be assigned to fibre-rich bran instead of wholegrains *per se* [14]. In addition to the beneficial health effects, however, dietary fibre may cause unwanted gastrointestinal effects such as flatulence, bloating, and abdominal discomfort [15]. In Finland, rye bread provides 30-50% of the total dietary fibre intake [16]. Consumption of rye bread has been shown to cause gastrointestinal symptoms to some but not to all individuals [17, 18]. The majority of fibre in rye is cell wall polysaccharides – cellulose, arabinoxylan, and ß-glucan – and fructan (a family of oligo- and polymers with degree of polymerization 3-60) [19, 20]. Fructan is more readily fermented than cell wall polysaccharides, and the fructan content of rye might explain the appearance of the gastrointestinal symptoms after rye intake [21]. Furthermore, the conventional rye bread in Finland is made of wholegrain flour with sourdough fermentation, which changes the nutritional quality and health effects of grain ingredients [22]. Sourdough wholegrain rye bread has a specific dense structure and sour taste, which may not appeal to consumers outside the Northern and Eastern Europe. In other parts of Europe, such as in Italy, refined grains are regarded as more tasty than wholegrains, and Italians and English perceive refined grains healthier than Finns do [23].

To increase the acceptability and to reduce fermentation-derived differences between wholegrain sourdough rye bread (WGR) and native rye bran, we baked a white wheat bread (WW) that was enriched with rye bran bioprocessed with enzymes and yeast (BRB) and contained the same fibre level as WGR. The aim was to investigate whether the white wheat bread enriched with bioprocessed rye bran (BRB + WW) promotes similar effects to WGR on glucose metabolism and plasma levels of SCFAs in healthy subjects with self-reported gastrointestinal symptoms. In addition, we tested whether responses to consumption of WGR and BRB + WW differed from that of low-fibre WW.

## Methods

### Subjects

**Figure 1:**
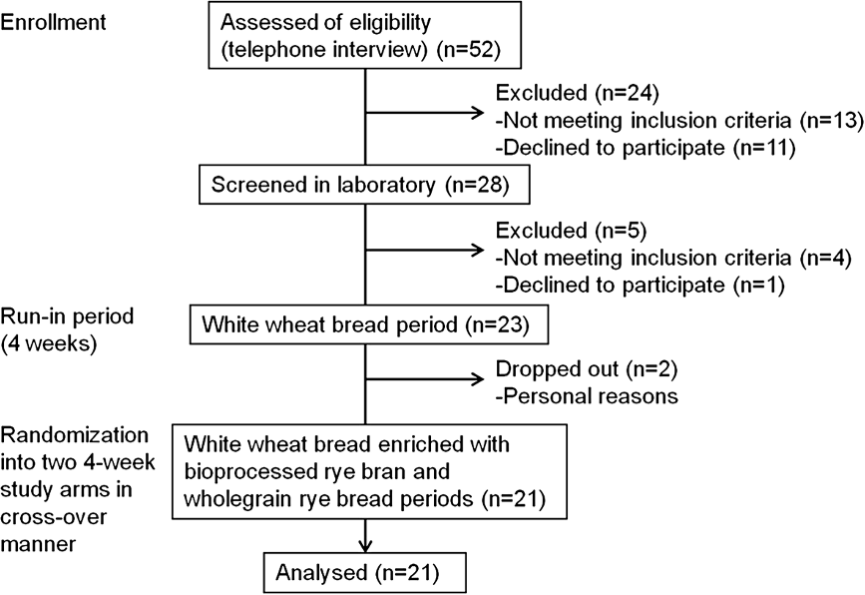
**Flow diagram.**

Healthy men and women were recruited based on one or several of the following self-reported gastrointestinal symptoms after ingestion of grain products, especially rye bread: flatulence, bloating, discomfort, constipation, and diarrhea. The recruitment of subjects is described in Figure 1. Exclusion criteria included BMI >35 kg/m^2^, inflammatory bowel disease, celiac disease or the presence of transglutaminase IgA antibodies (>7 U/ml), type 1 or 2 diabetes, abnormal liver, thyroid, or renal function (hyper- or hypothyroidism and hypertension controlled with medication were allowed), fasting serum triglycerides concentration >3.5 mmol/l, fasting serum total cholesterol concentration >8 mmol/l, alcohol abuse (>16 portions/week (women)/>24 portions/week (men)), cereal or milk protein allergy, special diet (such as vegetarian or low-carbohydrate diet), and antibiotic use over the preceding two months. The age, BMI, and fasting glucose of the subjects ranged from 38 to 65 years, from 19 to 30 kg/m^2^, and from 4.9 to 6.3 mmol/l, respectively. When assessed for eligibility, subjects were informed that participation in the intervention requires daily consumption of bread over three months. Five subjects mentioned that they had reduced consumption of grain products due to gastrointestinal symptoms. Other subjects did not report changes in their habitual consumption of grain products despite the symptoms. The subjects provided written informed consent prior participating in the study. The study was approved by the Ethics Committee of the Hospital District of Northern Savo.

### Test breads

Refined WW were two commercial breads with 100% white wheat flour (Vaasan Oy, Finland). For baking the WGR, wholegrain rye flour was fermented with Baker’s yeast and lactic acid bacteria (*Lb. brevis*, *Lb. plantarum*) for 22 hours at 30°C. The sourdough was mixed thoroughly at the beginning of fermentation but not during the process. The sourdough was used at the 50% of substitution level in baking. The bran for the BRB + WW was fermented with enzymes and yeast and the bread was baked as previously described [24]. The bread dough were left to rest for 20 min in 28°C and 75% relative humidity, mixing twice for two and four minutes during resting. The breads were prepared in 400 g dough pieces, proofed for 50 min in 35°C and 80% relative humidity, and baked in 225°C for 20 min, with 15 seconds steaming in the beginning.

BRB + WW and WGR had the same total fibre content (10%) and soluble fibre content (2.1%), whereas WW had lower total fibre content (3%). Furthermore, the BRB + WW and the WGR contained 0.7% and 1.8% fructan, 0.4% and 0.7% ß-glucan, and 2.0% and 1.3% soluble arabinoxylan, respectively. Bioprocessing of the rye bran and sourdough fermentation of the wholegrain rye flour reduced the starch content of BRB + WW and WGR by 23% and 32%, respectively. The BRB + WW, WGR, and WW breads provided 10.3, 7.3, and 9.1% protein, and 3.5, 0.6, and 3.5% fat, respectively.

### Study design

**Figure 2:**
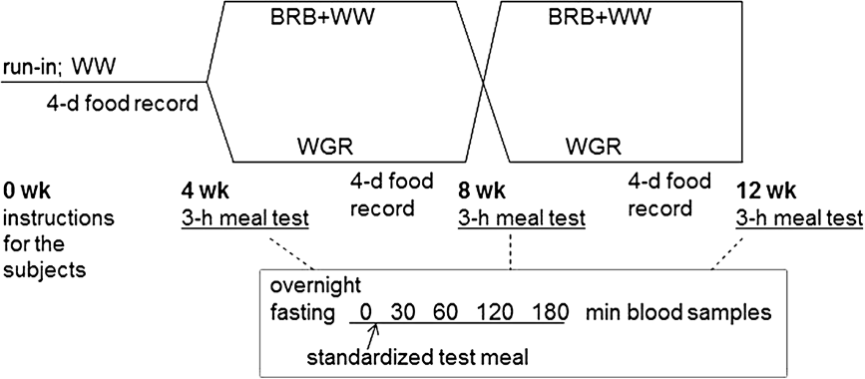
**Study design.** Test meal included a standardized portion of white wheat bread, margarine, cheese, cucumber, and juice. BRB + WW, white wheat bread enriched with bioprocessed rye bran; WGR, wholegrain rye bread; WW, white wheat bread.

A four-week run-in period with low grain fibre intake preceded two consecutive four-week test periods with high grain fibre intake in randomized, cross-over manner (Figure 2). During the run-in period the subjects were advised to consume 6-10 slices (20-25 g/slice) of the WW daily. During the test periods, the subjects were asked to consume 6-10 slices (25-30 g/slice) of the WGR and BRB + WW daily, in randomized order. The order of consuming the rye-containing products was randomized using the simple randomization method [25]. The specific amount of bread slices depended on individual energy requirement of the subjects. The test breads were provided for the subjects free of charge. The dietician advised the subjects weekly or biweekly in the practical management of the diet. Furthermore, the subjects were advised to maintain their body weight and follow their habitual living habits throughout the study.

For investigating gastrointestinal discomfort after consumption of the test breads, the subjects were required to exclude other food items possibly causing symptoms during the whole three-month intervention. Dietary counseling was based on avoiding vegetables, fruits, and pulses containing readily fermentable oligo-, di- and monosaccharides and polyols as well as foods supplemented with fructo-oligosaccharide, inulin, or galacto-oligosaccharide [21]. Instead, the subjects were asked to favor vegetables, fruits, and berries without or with only a low content of these fermentable carbohydrates. One to two small portions of other grain products than the test breads were allowed daily. If the subject was eating oatmeal daily during the run-in period, he/she was asked to keep this habit also during the other periods. If the subjects experienced constipation during the run-in period, occasional intake of non-grain fibre supplements such as dried and soaked plums, linseeds, and sugar beet fibre were allowed.

Subjects recorded the amount of the test breads and other grain products using a daily questionnaire, and filled in four-day food records during the last week of each period. The food records were analysed for nutrient intakes using Diet32 software (version 1.4.6.3, Aivo Finland Oy, Turku, Finland) which includes database of Finnish foods. Nutrient compositions of the WW, BRB + WW, and WGR were added to the database.

At screening and after each period, the subjects filled in a gastrointestinal quality of life questionnaire, GIQLI [26], which contained 36 questions on the gastrointestinal quality of life. The total score of GIQLI ranges from 0 to 144; the higher the score the better the gastrointestinal quality of life. The subjects also recorded the occurrence of specific gastrointestinal symptoms (flatulence, bloating, rumbling of stomach, abdominal pain, and heartburn) as well as defecation frequency daily over the first and last week of each period. The occurrence of symptoms was assessed by a 5-point scale as follows: 0 no symptoms; 1 slight symptoms; 2 moderate symptoms; 3 severe symptoms; 4 very severe symptoms.

### Standardized meal test

Instead of OGTT, we used a standardized meal test for experimental purpose to study glucose metabolism because it better reflects real life situation among free living individuals. The meal test was performed at the end of each four-week period. On the day preceding the meal test, the subjects were asked to avoid unusually large portions of food and avoid consumption of alcohol for two days before the meal test. After an overnight fast, the subjects arrived in the laboratory, their weight was measured, an intravenous catheter was inserted in the antecubital vein of the arm and a fasting blood sample was taken.

The meal included white wheat bread (80 g), milk-free margarine (20 g), cheese (20 g), cucumber (40 g), and juice concentrate (0.4 dl) diluted in 2.6 dl water. According to manufacturer’s information of each food item, the meal contained 550 kcal and 3.7 g fibre. Percentage of energy from carbohydrates, fat and protein was 51%, 33%, and 11%, respectively. Meal eating time was restricted to fifteen minutes. After starting the meal, four blood samples were taken at 30, 60, 120, and 180 minutes.

### Chemical and biochemical analyses

Chemical composition of the test breads regarding protein, fat, starch, total dietary fibre, soluble fibre and arabinoxylan, fructan, and ß-glucan was determined as described by Nordlund et al [27].

Blood samples were analysed for plasma glucose and insulin concentrations as follows: glucose was analysed using Konelab 20XTi Clinical Chemistry Analyser and Enzymatic photometric (glucose hexokinase) method (Konelab System Reagents, Thermo Fisher Scientific, Vantaa, Finland), and insulin was analysed with a chemiluminescent immunoassay (Advia Centaur Immunoassay System, Siemens Medical Solution Diagnostics, Tarrytown, NY, USA). Concentration of fasting serum total cholesterol and triglycerides were analysed using commercial kits (Thermo Electron Corporation, Vantaa, Finland) and Thermo Fisher Konelab 20XTi Analyser (Thermo Electron Corporation, Vantaa, Finland). To exclude celiac disease in screening, fasting serum sample was analysed by routinely used assay for transglutaminase IgA antibodies with fluoro-enzyme immunoassay method. SCFA in 0, 30, and 180 min plasma samples were measured by gas chromatography as described by Brighenti [28] with slight modifications using 2-ethyl butyrate (FLUKA no. 03190; Sigma Aldrich, St. Louis, MO, USA) as an internal standard instead of iso-valeric acid. The internal microbiota does not produce 2-ethyl butyrate, and it is consequently not present in biological samples.

### Calculations

Area under the curve (AUC) of glucose and insulin was calculated for each meal test from the area beneath the curve above the fasting level from 0 to 120 min using GraphPad Prism 4.0 for Windows (GraphPad Software, Inc., San Diego, CA, USA). First-phase insulin secretion, which is indicative of the insulin secretion capacity with relation to plasma glucose concentration, was calculated as following: (insulin 30 min – 0 min)/(glucose 30 min – 0 min). Disposition index (DI), which represents early insulin secretion taking insulin sensitivity into account, was calculated as a product of first-phase insulin secretion and insulin sensitivity (ISIcomp; [29]): (insulin 30 min – 0 min)/(glucose 30 min – 0 min)*(10000/√(G_0_*I_0_*G_M_*I_M_)) (G_0_ = fasting glucose concentration; I_0_ = fasting insulin concentration; G_M_ = mean of postprandial glucose concentrations; I_M_ = mean of postprandial insulin concentrations). Since there was no difference in the occurrence of gastrointestinal symptoms between the first and the last week of each period, the mean of the two weeks was calculated.

### Statistical analyses

To make the analysis of data on glucose and insulin responses at the meal test more similar to that the analysis of data from 2-hour OGTT, and because there were no statistically or clinically relevant differences in glucose, insulin, and SCFA concentrations at 180 min, results for glucose and insulin responses are reported based on 0, 30, 60, and 120 min plasma samples. The results for SCFA are reported based on 0 and 30 min plasma samples. First, glucose, insulin, and SCFA responses were compared after the WGR and BRB + WW periods. Then, the WW period was included in the analysis and the comparisons were made among the three periods. Comparisons between and among the periods regarding subjects’ characteristics, intake of nutrients, plasma glucose and SCFA concentrations, glucose AUC, first-phase insulin secretion, and DI were conducted using General linear model (GLM) for repeated measures. For GLM, non-normally distributed variables were logarithmic-transformed. However, plasma insulin concentrations, insulin AUC, gastrointestinal symptoms, frequency of defecation, energy intake, and total fibre intake were not normally distributed after logarithmic transformation, and thus were compared among the periods using the Friedman’s test and between the periods using the Wilcoxon signed rank test. All analyses were conducted with SPSS 19.0 for Windows (Chicago, IL). Because of the experimental purpose of the meal test, p-values for markers of glucose and insulin metabolism are reported as non-adjusted values when the comparisons were made among the three periods. For other variables, the p-values were corrected for multiple comparisons. P-values <0.05 were regarded statistically significant. The data is expressed as mean ± SD or mean ± SEM.

## Results

### Compliance and tolerance to diet

The subjects consumed the test breads as advised based on the daily questionnaires. The intake of energy and percentage of energy from fat did not change during the intervention, whereas the percentage of energy from protein was slightly higher during the BRB + WW period as compared to the other periods (p < 0.05) (Table 1). The percentage of energy intake from carbohydrates was lower (p < 0.001) during the BRB + WW period than during the other periods due to the lower starch content of the bread. The intake of total fibre and fibre from the breads was lower during the WW period than during the other periods (p < 0.001), and similar between the BRB + WW and WGR periods. Consumption of other grain products was similar over each period (1.0 ± 0.6 portions/d). Total serum cholesterol was significantly (p < 0.01) higher at the end of BRB + WW than WGR period (Table 2).

**Table 1:** **Intake of energy, nutrients, and test breads over the bread periods (n = 21)**

	WW period	BRB + WW period	WGR period	p-value ^1^
Energy, kJ/d (kcal/d)	8 620 ± 1 720 (2 060 ± 410)	8 410 ± 2 140 (2 010 ± 510)	8 210 ± 1 930 (1 960 ± 460)	0.405
Carbohydrates, E%	43 ± 6	38 ± 7***	42 ± 7^###^	< 0.001
Protein, E%	20 ± 3	22 ± 3*	20 ± 3^#^	0.003
Fat, E%	33 ± 5	35 ± 6	32 ± 6	0.094
Total fibre, g/d	21 ± 7	34 ± 10***	33 ± 10***	< 0.001
Bread, g/d	169 ± 24	195 ± 53	205 ± 50***	< 0.001
Fibre from bread, g/d	5 ± 1	20 ± 5***	21 ± 5***	< 0.001

^1^Among the bread periods (GLM for repeated measures adjusted for multiple comparisons; for energy and total fibre Friedman’s test followed by Wilcoxon singed rank test adjusted for multiple comparisons). Different from WW period: *p < 0.05, ***p < 0.001. Different from BRB + WW period: ^#^p < 0.05, ^###^p < 0.001. BRB + WW, white wheat bread enriched with bioprocessed rye bran; E%, percentage of total energy intake; WGR, wholegrain rye bread; WW, white wheat bread. Data are presented as mean ± SD.

**Table 2:** **Characteristics of the subjects at end of each bread period (n = 21)**

	WW period	BRB + WW period	WGR period	p-value ^1^
Weight (kg)	72 ± 14	72 ± 13	72 ± 14	0.133
Fasting glucose (mmol/l)	5.1 ± 0.4	5.0 ± 0.5	5.0 ± 0.5	0.449
Cholesterol (mmol/l)	5.2 ± 0.9	5.4 ± 0.9	5.0 ± 0.7^##^	0.006
Triglycerides (mmol/l)	0.9 ± 0.4	0.9 ± 0.4	1.0 ± 0.6	0.499

^1^Among the bread periods (GLM for repeated measures adjusted for multiple comparisons). Different from BRB + WW period: ^##^p < 0.01. BRB + WW, white wheat bread enriched with bioprocessed rye bran; WGR, wholegrain rye bread; WW, white wheat bread. Data are presented as mean ± SD.

At screening, the GIQLI score was 119 ± 12, while at the end of the run-in, WGR and BRB + WW periods the score was 127 ± 9, 125 ± 10 and 126 ± 9, respectively. Thus, the gastrointestinal quality of life of the subjects was significantly better (p < 0.01) over the 4-week run-in and test periods than at the screening when they followed their habitual diet. Based on the questionnaire on the occurrence of the specific gastrointestinal symptoms the subjects reported slight to moderate flatulence more frequently over the BRB + WW and WGR periods than over the WW period (p < 0.05). Altogether 24% (n = 5) and 29% (n = 6) of the subjects reported moderate or severe flatulence over BRB + WW and WGR periods, respectively, while only one subject reported moderate or severe flatulence during the WW period. There were no reported differences in bloating, rumbling of stomach, abdominal pain, or heartburn among the periods, and over 90% of the subjects reported none or slight symptoms. Frequency of defecation was not affected by the period, being on average 1.4 times per day.

### Glucose and insulin

**Figure 3:**
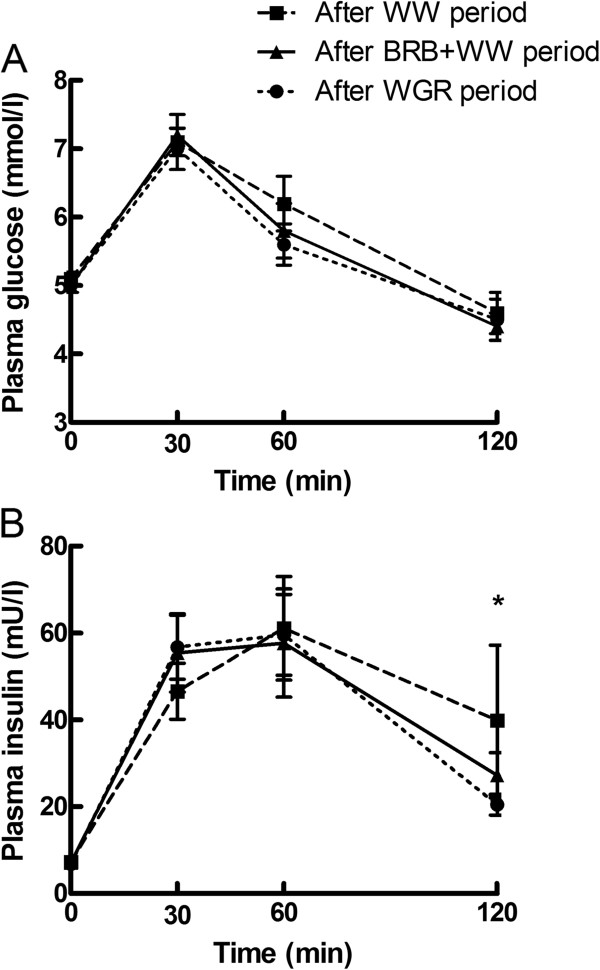
**Fasting and postprandial A) glucose and B) insulin responses to the meal test following the four-week periods with consumption of the different test breads (mean ± SEM, n = 21).** *p < 0.05 between the WGR and WW periods (Wilcoxon signed rank test). ▲, BRB + WW, white wheat bread enriched with bioprocessed rye bran; ●, WGR, wholegrain rye bread; ▀, WW, white wheat bread.

Fasting and postprandial glucose and insulin responses to the meal test did not differ between the WGR and BRB + WW periods (Figure 3). No difference was found in glucose and insulin AUCs and the first-phase insulin secretion (data not shown), and in the disposition index (DI) (3519 ± 4947 and 3614 ± 2883 for BRB + WW and WGR periods, respectively) between the periods. However, response of plasma insulin to the meal test was lower after the WGR period than after the WW period at 120 min (p = 0.023, Wilcoxon test) (Figure 3B). On the other hand, first-phase insulin secretion during the meal test tended to be higher after the rye bread periods (p = 0.083, GLM). Also, DI differed among the bread periods (p = 0.042, GLM), being higher after the WGR period as compared to the WW period (3614 ± 2883 vs. 2500 ± 1336, p = 0.033, Wilcoxon).

### Plasma SCFA

**Figure 4:**
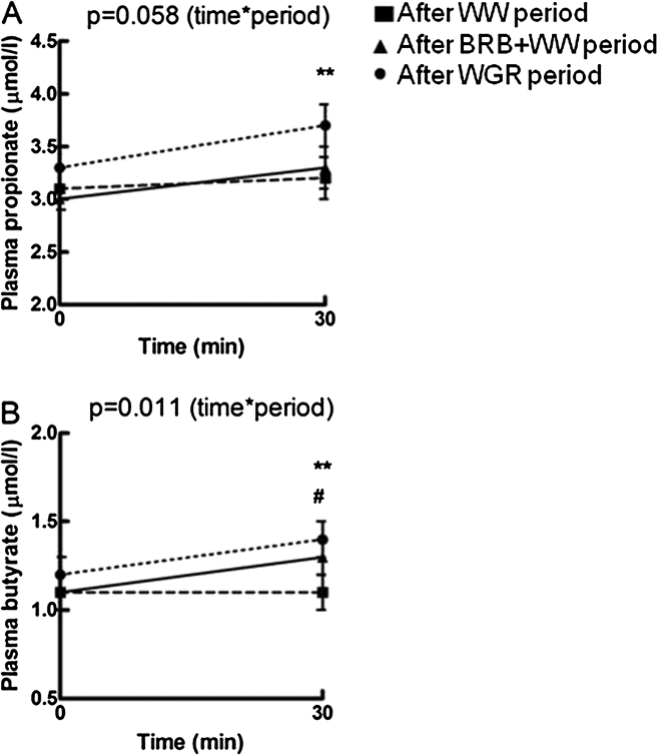
**Fasting and postprandial A) propionate and B) butyrate concentrations in response to the meal test following the four-week periods with consumption of the different test breads (mean ± SEM, n = 21).** ▀, after WW period; ▲, after BRB + WW period; ●, after WGR period. *p < 0.05, **p < 0.01 between the WGR and WW periods, #p < 0.05 between the BRB + WW and WW periods (General linear model for repeated measures adjusted for multiple comparisons). BRB + WW, white wheat bread enriched with bioprocessed rye bran; WGR, wholegrain rye bread; WW, white wheat bread.

Fasting concentration of total SCFAs (sum of acetate, propionate, and butyrate mean ± SD of all periods: 89 ± 26 μmol/L) and of individual SCFAs did not differ among the periods. Acetate concentration accounted for 94% of the total concentration of SCFAs. From fasting to 30 min postprandially, the total SCFA, acetate, propionate, and butyrate concentrations were similar between the WGR and BRB + WW periods. However, when comparisons were made among all the three periods, propionate concentration tended to differ (p = 0.058, GLM for time x period interaction) and butyrate concentration differed significantly (p = 0.011, GLM for time x period interaction) from fasting to 30 min postprandially (Figure 4A,B). At 30 min, propionate concentration was higher after the WGR period than after the WW period at 30 min (p < 0.01, Wilcoxon test), and butyrate concentration was higher after the WGR (p < 0.01, Wilcoxon test) and BRB + WW periods (p < 0.05, Wilcoxon test) than after the WW period.

## Discussion

In the present study, we compared the effects of four-week consumption of WGR and BRB + WW on postprandial glucose and insulin responses and plasma concentrations of SCFA after a standardized meal test. Enrichment of white wheat bread with bioprocessed rye bran (BRB + WW) produced nearly similar effects than WGR on glucose metabolism and plasma concentrations of SCFAs, although the effects of WGR were more pronounced. When compared to the WW bread period, both rye bread periods increased the release of butyrate and tended to increase the first phase insulin response, while reducing the insulin concentration at 120 min after the test meal. The results suggest that the first-phase insulin secretion is improved after consuming rye breads and less insulin is needed in the later postprandial phase to control glucose concentration. Improved first-phase insulin secretion and subsequent reduced hyperinsulinemia in later postprandial phase may prevent disturbances in glucose metabolism [30].

BRB + WW included white wheat flour in addition to rye bran, and had the same fibre content as WGR which was baked from wholemeal rye flour. The equal fibre content might explain the similar effects. However, rye bread given as a single meal may have additional effects that are independent of the total fibre content of bread. A single meal of rye bread has been shown to reduce postprandial insulin response in healthy subjects as compared to that of white wheat bread, and this effect is not related to the fibre content of the rye bread [2–4]. In the present study WGR provided 2.5-fold more fructan (3.7 vs. 1.4 g/d) than BRB + WW bread, but inulin-type fructans do not seem to affect fasting glucose concentration in humans [31], and there is no evidence that >2 g more fructan per day would affect insulin metabolism. Sourdough fermentation does not seem to play a role in this effect since also endosperm and wholegrain rye breads baked without sourdough fermentation process reduce the acute postprandial insulin response [2]. Furthermore, a single meal containing BRB + WW bread did not reduce the insulin response in our recent postprandial study [24], indicating that BRB + WW does not have similar acute effects on glucose metabolism as WGR.

We observed improved first-phase insulin secretion after consuming WGR as compared to that of WW when possible variation in insulin sensitivity between the subjects was taken into account. To our knowledge we are the first who have used a standardized meal test to investigate glucose metabolism after a longer-term regular intake of rye bread. Some earlier intervention studies have failed to show effects by rye bread on fasting glucose and insulin concentrations [4, 6, 32, 33] or on specific parameters of glucose and insulin metabolism measured by FSIGT [7], as compared to refined wheat bread. However, consumption of rye bread-based diet has been reported to improve the first-phase insulin secretion in OGTT as compared to the wheat-based control diet [5], and an improvement in the first-phase insulin secretion in OGTT has been observed in subjects with normal glucose tolerance after consumption of sourdough wholegrain wheat bread vs. white wheat bread [34]. Methodological differences and differences in study populations may explain the inconsistencies in these results. It was postulated that the effects of wholegrains on insulin sensitivity may be mediated by gastrointestinal hormones secreted postprandially after a meal [7], a condition which is not met in the FSIGT. Unlike FSIGT, OGTT takes into account the postprandial events, but in OGTT only glucose is ingested and no digestion is required. On the contrary, the standardized meal test, as used in the present study, mimics the true physiological event of ingestion of a mixture of foods that are digested to nutrients and absorbed and may differently affect gastrointestinal hormones.

The increase in fasting cholesterol due to consumption of the bread containing bioprocessed rye bran may be explained by slightly, although not statistically significant, higher intake of dietary fat during the BRB + WW period. However, this requires further investigation, because similar effect was observed in postmenopausal women consuming a diet containing rye bread enriched with rye bran and maintaining fat intake unchanged [35].

We observed no differences in concentrations of total and individual SCFAs in fasting plasma between the rye-containing and WW diets, supporting the previous finding [36] that consuming high-fibre rye bread does not seem to affect fasting plasma level of SCFAs in humans. However, rapid increase in the concentration of plasma butyrate after the standardized meal was observed following the rye bread periods, while WGR increased also the concentration of propionate as compared to WW. Increased butyrate concentrations after rye containing diets have been observed in human faeces [33, 37] and the portal vein [38, 39], and in the peripheral blood of pigs [36]. We propose that it is possible to observe the effects of intestinal fermentation on plasma concentrations of SCFA in humans when measured in the postprandial state. The finding that plasma SCFA concentration increases rapidly during 30 minutes after the standardized test meal is similar as detected in previous studies [8, 10, 12]. However, the reason for this increase is currently not known, but most likely the increase reflects the metabolism of fibre components from the previous meals, not from the standardized meal itself.

The improvement in postprandial insulin responses during the WGR period appeared to occur simultaneously with increase in plasma concentrations of propionate and butyrate. This finding is in line with the second meal studies showing an increase in plasma propionate and/or butyrate concentrations simultaneously with reduction in postprandial glucose response after a standardized breakfast following an evening meal with barley [8, 10, 12]. Feeding rye bread to pigs increased absorption of butyrate from the intestine and lowered insulin response, suggesting improved insulin sensitivity either at the liver level or in muscles [39]. Increase in insulin sensitivity has also been associated with increase in butyrate producing intestinal bacteria in subjects with metabolic syndrome [40]. However, our study does not unambiguously support the previously observed associations between butyrate and glucose metabolism, because also consumption of the BRB + WW increased plasma concentration of butyrate at 30 min without significant improvement in the postprandial insulin responses as compared to WW. On the other hand, we cannot rule out that increased concentration of plasma propionate after consumption of WGR could improve glucose metabolism.

In our study, self-reported gastrointestinal symptoms seemed not to limit the consumption of the rye-containing breads. Surprisingly, subjects’ gastrointestinal quality of life improved during the controlled consumption of the test breads. This might be due to avoidance of foods containing readily fermentable carbohydrates, i.e. certain vegetables, fruits, pulses, and fibre-supplemented food products [21]. It is suggested that intake of the rye-containing breads, where most of the fermentable carbohydrates exist as cell wall polysaccharides, is tolerated better when the total load of the readily fermentable carbohydrates is reduced. It is noteworthy that the rye-containing breads did not decrease subjects’ quality of life despite causing flatulence.

## Conclusions

In conclusion, our findings confirm the beneficial effects of WGR over white wheat bread on glucose and SCFA production. Also, the enrichment of the white wheat bread with bioprocessed rye bran yielded similar but not as pronounced effects than WGR when compared to WW alone. We hereby present, in a proof of concept manner, the potential of making healthier alternatives to white wheat in more widely acceptable and technologically utilizable form than WGR. Furthermore, the study emphasizes the importance of postprandial measurements of glucose metabolism and plasma concentrations of SCFA in addition to fasting measurements.
